# Effect of electro-acupuncture on lateralization of the human swallowing motor cortex excitability in healthy subjects: study protocol for a single-blind, randomized controlled trial

**DOI:** 10.1186/s13063-019-3267-x

**Published:** 2019-03-21

**Authors:** Minying Li, Lin Wang, Nenggui Xu, Xiaorong Tang, Mindong Xu, Jianhua Liu, Jianpeng Huang, Judith M. Schlaeger

**Affiliations:** 1 South China Research Center for Acupuncture and Moxibustion, Medical College of Acu-Moxi and Rehabilitation, Guangzhou University of Chinese Medicine, Guangzhou, 510006 China; 2 Traditional Chinese Medicine Hospital of Guangdong Province, Guangzhou, 510000 China; 3 Department of Women, Children and Family Health Science, University of Illinois at Chicago College of Nursing, 845 S. Damen Ave. (M/C 802), Chicago, IL 60612 USA

**Keywords:** Electro-acupuncture, Lateralization of the swallowing motor cortex, Dysphagia, Stroke, Transcranial magnetic stimulation

## Abstract

**Background:**

Numerous randomized controlled trials on the effects of electro-acupuncture have been conducted to treat dysphagia as a sequela of stroke. However, the normal physiological mechanisms of swallowing and the pathological mechanisms of dysphagia are not fully understood. The purpose of this study is to investigate whether lateralization of the human swallowing motor cortex excitability in healthy subjects will be influenced by electro-acupuncture to Lianquan (CV 23) and Fengfu (GV 16), which may provide insight into the pathological mechanisms of dysphagia after stroke.

**Methods:**

We designed a single-blind, randomized, sham-controlled trial in which 40 healthy subjects will be recruited. Subjects will be randomized 1:1 into two groups: the electro-acupuncture group and the sham-control electro-acupuncture group. The swallowing motor cortex will be located in both groups using a neuroimaging navigation system. Then left and right cortical stimulation will be measured by transcranial magnetic stimulation (TMS) before and after electro-acupuncture or sham electro-acupuncture. The electro-acupuncture or sham electro-acupuncture interventions will last for 15 min. The primary outcome measure will be percent change in the resting motor threshold (RMT) of the mylohyoid. The secondary outcome measures will be the amplitude (μV) and latency (ms) of the motor evoked potential (MEP) of the mylohyoid as a proxy for the TMS evoked potential. All outcomes will be measured at baseline and after the electro-acupuncture or sham electro-acupuncture treatment.

**Discussion:**

The aim of this trial is to explore whether lateralization of the human swallowing motor cortex excitability in healthy subjects is present, and to determine if electro-acupuncture to acupuncture points Lianquan (CV 23) and Fengfu (GV 16) will exert an effect on it under normal physiological conditions.

**Trial registration:**

Chinese Clinical Trial Registry, ChiCTR-IOR-17011359. Registered on 11 May 2017.

**Electronic supplementary material:**

The online version of this article (10.1186/s13063-019-3267-x) contains supplementary material, which is available to authorized users.

## Background

The main role of swallowing is to transport food and drink from the oropharynx to the stomach while preventing aspiration [1]. Healthy adults swallow approximately 800 to 2400 times per day. Swallowing is a complex process comprised of five distinct phases [2]. Research in humans has demonstrated that automatic and volitional swallowing are under the control of the human cerebral cortex and are associated with activation of several spatially and functionally distinct cortical regions [3]. Oropharyngeal dysphagia (OD) is a common sequela of stroke that occurs if any phase in the swallowing process is disrupted. To fully understand the sensory motor functions of swallowing in the pathological state (as a sequela of stroke in patients with OD), lateralization of the human swallowing motor cortex must be examined in the normal physiological state in healthy subjects. Overall, 27% of the elderly population has OD which affects 47.5% of the hospitalized elderly (of those up to 91% have community-acquired pneumonia) and 51% of nursing home residents [4–9]. Dysphagia as a sequela of stroke potentiates serious health conditions such as aspiration, aspiration pneumonia, and malnutrition, which may lead to depression, anxiety, decreased quality of life, and death [10].

Lateralization of the functions and structures of the human and animal brain has been known since the 1860s when the French surgeon Paul Broca discovered that the two cerebral hemispheres have different functions [11]. This important finding established the foundation for research examining pathological processes in the brain regions involved in stroke, particularly the cerebral hemispheres [12, 13]. Scientists then began to examine cerebral hemispheric lateralization in healthy subjects under normal physiological conditions to uncover the mechanisms involved in this process. In the last 20 years, researchers worldwide have examined the swallowing motor cortex and its role in diseases that present with dysphagia using transcranial magnetic stimulation (TMS) in healthy subjects as well as in subjects with dysphagia as a sequela of cerebral hemisphere stroke. Whether patients suffer from dysphagia after a cerebral hemisphere stroke depends on the swallowing motor cortex map size in the undamaged cerebral hemisphere. Also, recovery from dysphagia is associated with the reorganization of the swallowing motor cortex in the undamaged cerebral hemisphere [14–17]. Increasing sensory input to the motor cortex via electrical stimulation results in an increased ability to swallow and hastens rehabilitation after stroke. The purpose of this study is to investigate whether lateralization of the human swallowing motor cortex excitability in healthy subjects under normal physiological conditions will be influenced by electro-acupuncture. Results may provide insight into the pathological mechanisms of dysphagia after stroke.

A 2017 systematic review of 71 studies, with over 6000 subjects enrolled, examined the efficacy of acupuncture for the treatment of OD as a sequela of stroke. Findings indicated that: 1) in a pooled analysis, acupuncture had a statistically significant effect compared with the controls, with moderate heterogeneity (*p* < 0.0001); and 2) the efficacy rate of acupuncture was three times that of the controls, demonstrating that acupuncture was efficacious for the treatment of dysphagia after stroke [18].

Electro-acupuncture has been used to treat dysphagia as a sequela of stroke to enhance swallowing ability [19]. One study used a microcurrent device that emitted a weak electrical signal of less than 1 μA attached with electrodes to acupuncture needles. Findings showed that electro-acupuncture produced a more widespread signal and therefore may be more effective than manual acupuncture [20].

After examination with TMS, acupuncture to Hegu (LI 4) demonstrated a statistically significant reduction in right hemisphere motor cortex excitability (*p* = 0.048) and an increase in left hemisphere motor cortex excitability which was not statistically significant (*p* = 0.16) [21]. Also, acupuncture to right Yanglingquan (GB 34) decreased the excitability of the left hemisphere motor cortex (*p* = 0.047) [22]. Both these studies demonstrated that acupuncture administered to acupuncture points on the extremities can modulate the lateralization of motor cortex excitability and provide the necessary foundation to study lateralization of the swallowing motor cortex excitability with electro-acupuncture.

Lianquan (CV 23) is an acupuncture point on the anterior of the surface of the neck directly superior to the laryngeal prominence, in the depression at the upper margin of the hyoid bone. It is at the base of the tongue and level with the pharynx. CV 23 is also located where the mylohyoid (which is a paired muscle running from the hyoid bone to the mandible and which forms the floor of the mouth) inserts into the hyoid. The mylohyoid is responsible for swallowing and speaking.

Fengfu (GV 16) is 1 cun directly above the midpoint of the posterior hairline in the depression below the external occipital protuberance. GV 16 is just ventral and rostral to the medulla oblongata (the center of deglutition) which controls swallowing [23].

Plasticity of the swallowing motor cortex is imperative for the recovery of patients with OD after stroke. It requires stimulation from sensory input and an undamaged map area. Studies examining the mechanisms of electro-acupuncture on OD as a sequela of stroke are difficult to perform. Therefore, lateralization of the human swallowing motor cortex excitability with electro-acupuncture in healthy subjects must be performed. The aim of this trial is to explore whether lateralization of the human swallowing motor cortex excitability in healthy subjects is present. We will then investigate the effects of electro-acupuncture on lateralization of the human swallowing motor cortex excitability by recording the resting motor threshold (RMT) and the motor evoked potential (MEP) changes in an electro-acupuncture and a sham electro-acupuncture group. We hypothesize that after administering electro-acupuncture to healthy subjects there will be a decrease in the RMT and an increase in the amplitude of the MEP in the swallowing motor cortex. This will demonstrate the activation of the swallowing motor cortex by electro-acupuncture. Results of this study will both help identify the swallowing mechanism in healthy subjects and enhance the understanding of the pathological mechanisms of OD as a sequela of stroke. It is possible the other undamaged hemisphere map area may be activated by electro-acupuncture as well, which may ultimately demonstrate mechanisms of cerebral functional reorganization and cerebral plasticity in OD as a sequela of stroke.

## Methods/design

### Study design

**Fig. 1:**
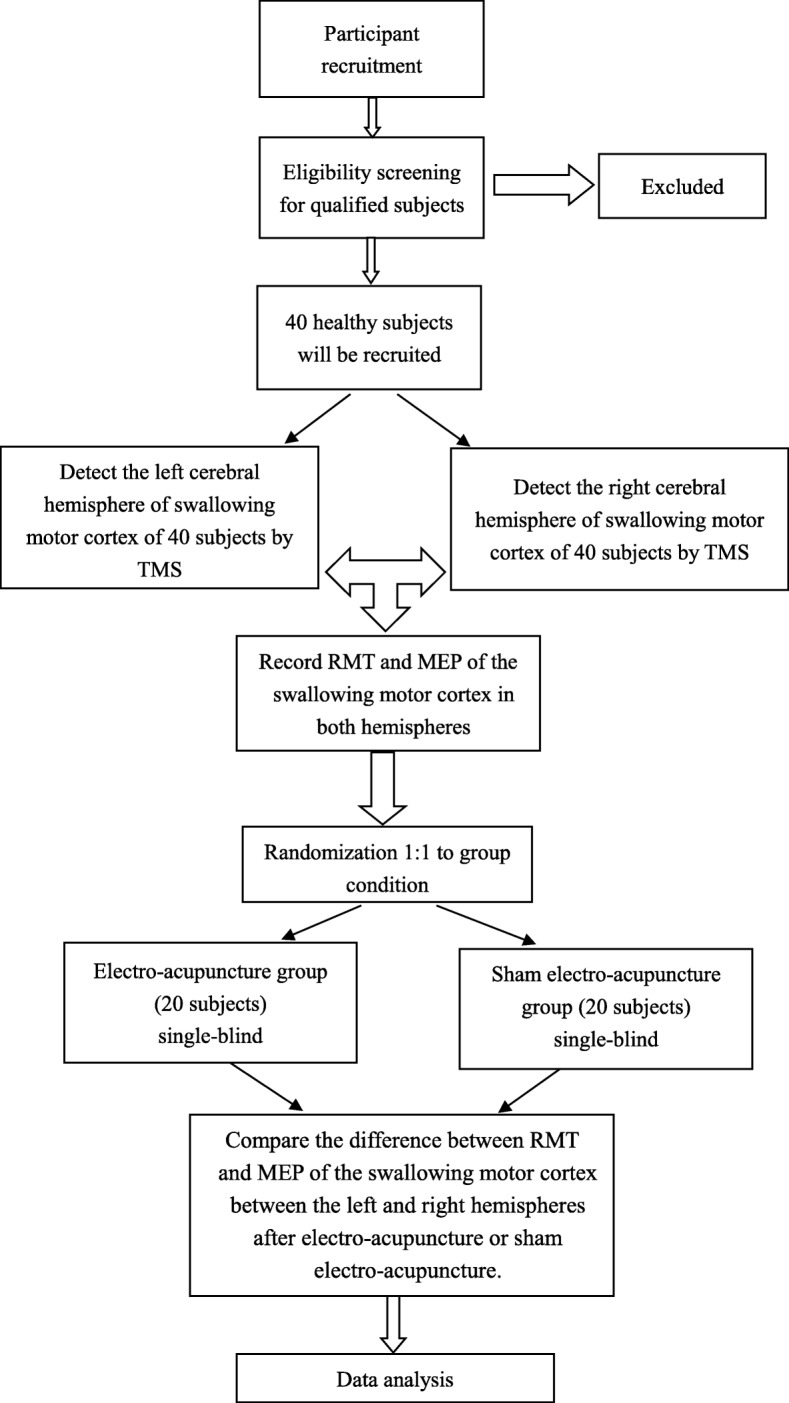
Consort flow chart. MEP motor evoked potential, RMT resting motor threshold, TMS transcranial magnetic stimulation

This will be a single-blind, randomized controlled trial (RCT). Electro-acupuncture will be compared with sham electro-acupuncture to determine if electro-acupuncture has an effect on lateralization of the human swallowing motor cortex excitability. Forty healthy subjects will be blinded to the study condition. This RCT will be conducted at the South China Research Center for Acupuncture and Moxibustion from April 2017 to April 2019. This trial has been registered with the Chinese Clinical Trial Registry (ChiCTR-IOR-17011359). The study design is presented in the flowchart in Fig. 1. The Standard Protocol Items: Recommendations for Interventional Trials (SPIRIT) checklist is provided as Additional file 1.

### Recruitment

The participants will be recruited by posting study recruitment flyers in classrooms at Guangzhou University of Chinese Medicine. Participants will contact the recruitment staff by telephone or email. Recruitment staff will be responsible for the enrollment of participants. If they meet the study criteria, they will be invited to the South China Research Center for Acupuncture and Moxibustion to undergo the study.

### Inclusion criteria

Participants who meet the following inclusion criteria will be eligible to participate in this study: 1) male or female; 2) between 18 and 25 years of age; 3) have no history of health problems, swallowing disorders, or pain with swallowing; 4) have a negative Kubota Water Swallow Test (test for OD); 5) have a normal Mini-Mental State Examination (MMSE) (a measure of cognitive impairment); 6) have a body mass index (BMI) between 18.5 and 24 kg/m^2^; and 7) be willing to sign the informed consent form.

### Exclusion criteria

Subjects will be excluded if they have any of the following conditions: 1) a history of major diseases, surgeries, or severe traumas; 2) swallowing disorders of any etiology; 3) presence of laryngeal organic diseases such as severe pharyngitis, rhinitis, or upper respiratory infection, nasal polyps, or deviated nasal septum; 4) difficulty breathing; 5) presence of viral hepatitis, HIV, or other blood infections; 6) presence of a cardiac pacemaker or other implanted devices such as brain metal electrodes (except titanium), cochlear implants, or pulse generators; 7) conditions that may potentiate seizures such as a history of epilepsy, sleep deprivation, alcohol dependence, or ingestion of olanzapine or lithium carbonate; and 8) pregnancy or lactation.

### Randomization and allocation concealment

Forty healthy subjects will receive TMS bilaterally on both swallowing motor cortices. Then they will be randomized 1:1 to an electro-acupuncture group or sham electro-acupuncture group. Randomization will occur according to a computer randomization program generated by the Package for Encyclopedia of Medical Statistics (PEMS) 3.1 software (West China School of Public Health, Sichuan, China) and overseen by an independent statistician. Subjects will be sequentially assigned a random number from 1 to 40. Opaque sealed envelopes will be used for the randomization and allocation. The envelopes will be numbered consecutively with a serial number on the outside and will contain the allocation information. An envelope will be opened when a subject enters the study after informed consent is obtained. Participants will be allocated to one of the two groups and receive an intervention according to their group allocation. The random allocation sequence and the opaque sealed envelopes will be kept by two different researchers.

### Blinding

Due to the specific nature of acupuncture, it is difficult for acupuncture researchers to be blind to the study condition. Consequently, in order to maintain blinding the subjects, researchers and the statistician will be blind to the study condition, but not the acupuncturist. The data will be analyzed by two statisticians who will otherwise not be involved in this trial and are naive to all study processes.

### Study procedures

Subjects consented to the study will complete the MMSE, Kubota Water Swallowing Test, and be weighed so that BMI may be calculated in an examination room at the South China Research Center for Acupuncture and Moxibustion, Medical College of Acu-Moxi and Rehabilitation, Guangzhou University of Chinese Medicine. They will sit on the treatment couch facing the neuroimaging navigation system and TMS machine to enable location of the correct brain regions. Subjects will be instructed not to swallow, cough, or speak when receiving TMS as these actions will cause artifacts and render the results invalid.

Surface electrodes will be placed on the skin over the mylohyoid after being sanitized with skin scrub. Mylohyoid electromyography (EMG) responses will be detected using two pairs of bipolar silver-silver chloride electrode (10 mm diameter, 1.5 m cable 12/package, DIN Style, Nicolet, USA); the surface record electrodes will be placed bilaterally on each side of the mylohyoid, and the reference electrodes will be placed directly next to them (1 cm). Two pairs of ground wire disc electrodes (1. 25 m cable, 1/package, DIN Style) will be placed 2 cm from both corners of the mouth to reduce interference to the EMG signal. All electrodes will be checked every 15 min to ensure that they are in contact with the skin and underlying muscles.

### Navigation

**Fig. 2:**
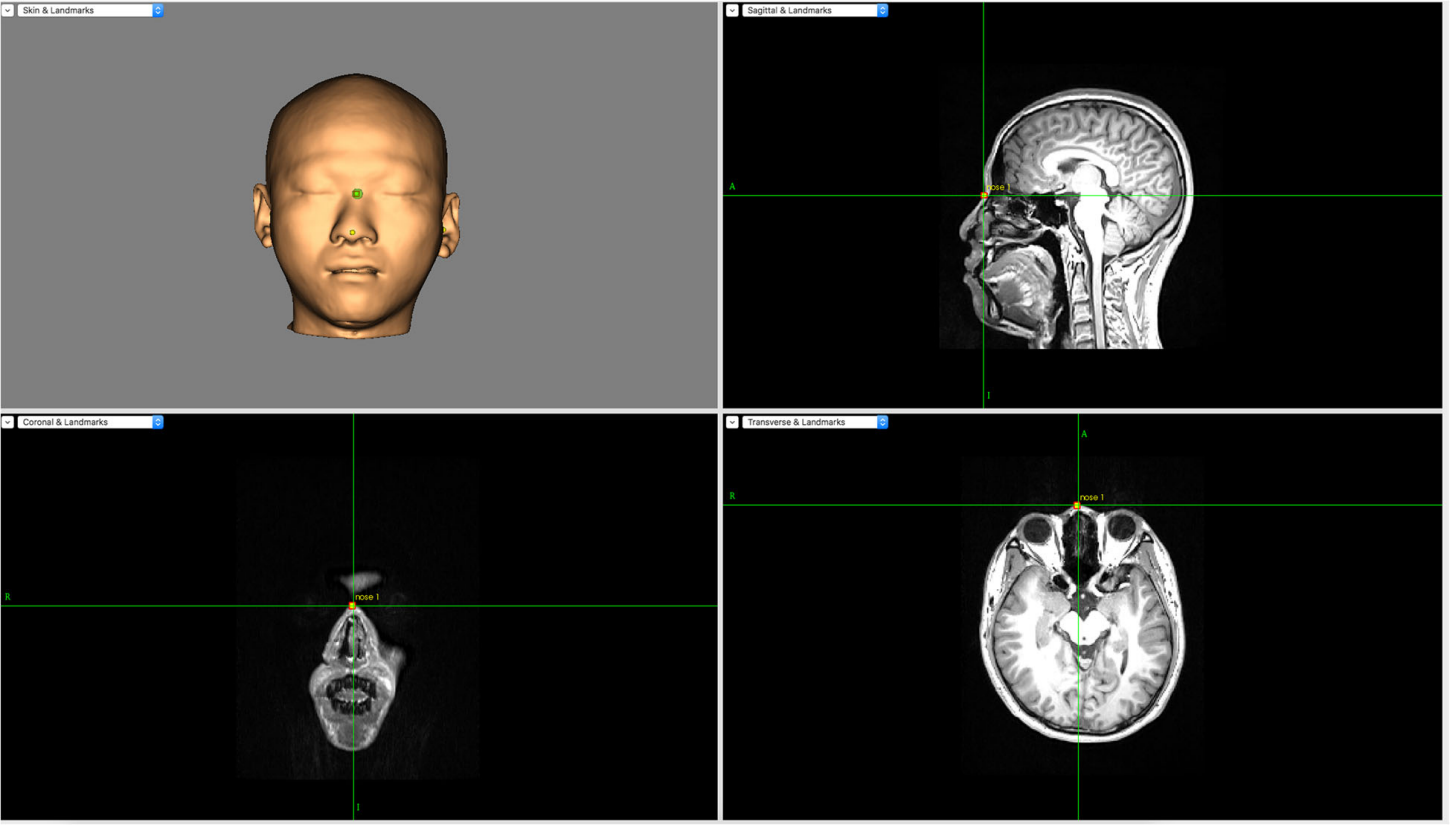
Three-dimensional brain reconstruction

**Fig. 3:**
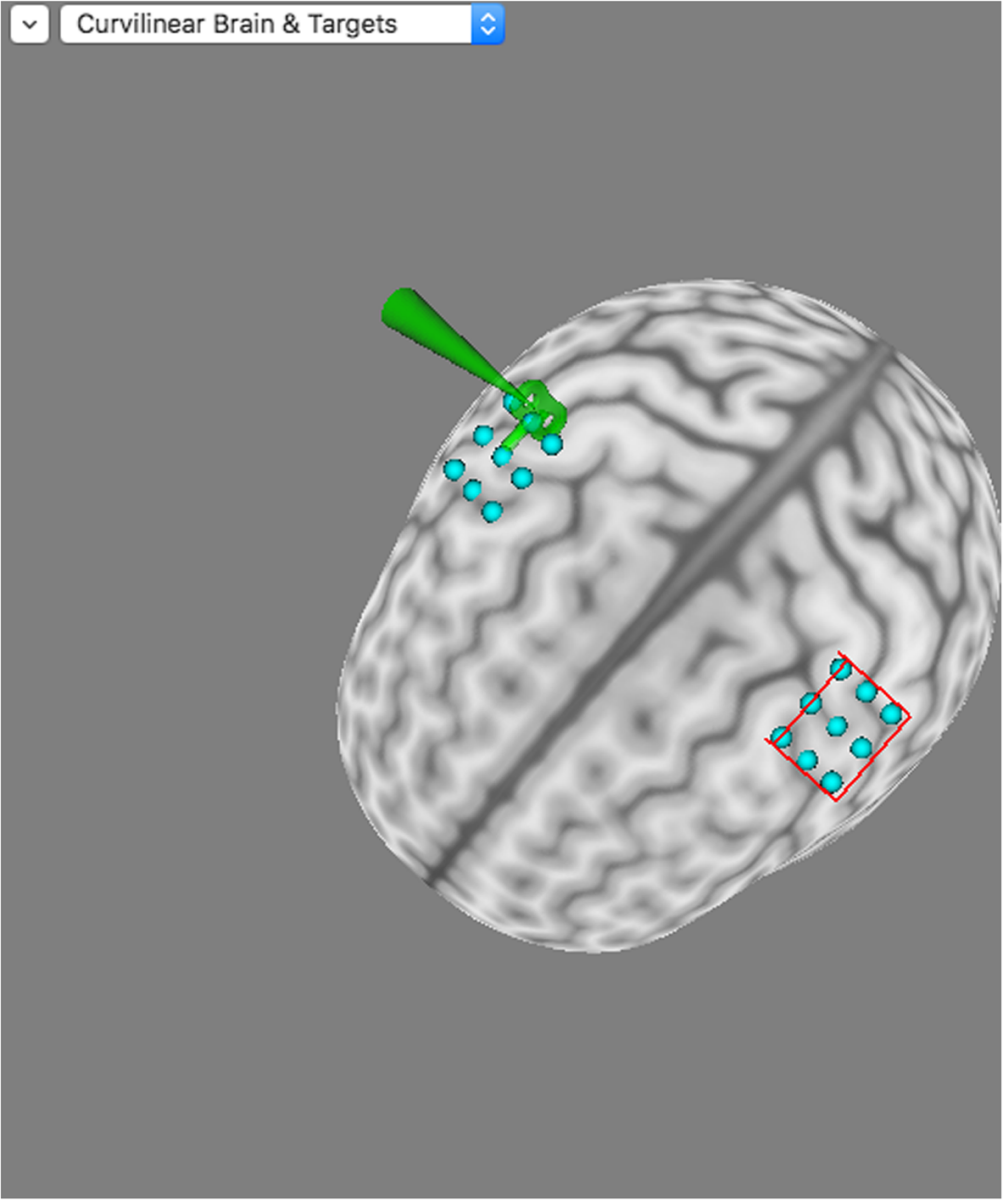
Neuroimaging navigation of the swallowing motor cortex

The subject’s magnetic resonance image (MRI) (3.0 T) T1 file will be scanned at Guangdong Provincial Hospital of Chinese Medicine with an MRI scanner (signa EXCITE 3.0 T HD, IGE, USA). This MRI T1 file will be imported into the neuroimaging navigation system (Brainsight^*®*^ 2.3.3.dmg). Brainsight^®^ will then be applied to reconstruct a three-dimensional (3D) brain. The reconstruction is shown in Fig. 2. The Polaris System will be used to collect cortical topographical landmarks of the head, which allows the external near infrared system to follow in real time the stimulator coil of the TMS. An image of a swallowing motor cortex navigation is shown in Fig. 3.

### Transcranial magnetic stimulation

Transcranial cortical stimulation will be performed at each session using a commercially available magnetic stimulator that is connected to a figure-of-eight coil before and after the intervention. TMS allows focal stimulation to areas of the cerebral cortex up to a maximum of 2.2 T.

### Intervention

Electro-acupuncture or sham electro-acupuncture will be performed for one 15-min session by an experienced doctor of acupuncture. A sterile acupuncture needle (length 25 mm, diameter 0.30 mm; Huatuo, Suzhou Medical Supply Factory Co. Ltd., Suzhou, China) will be inserted into CV 23 to a depth of 0.5 to 0.8 cun, and into GV 16 to a depth of 0.5 to 1 cun. The researcher will then apply electro-acupuncture by connecting an acupuncture point nerve stimulator (HANS-200A) to the needles with a frequency of 2 Hz for 15 min; the intensity of electro-acupuncture will be set according to the maximum tolerated intensity of each subject (between 0.9 mA and 3 mA) (Wang, Lin. Effect of Electroacupuncture at Acupoints on the Conception Vessel and Governor Vessel on the Swallowing Motor Cortex in Humans, unpublished). To maintain study fidelity, all researchers will receive training on the study methods, techniques, and study monitoring. All research staff will be tested after training to ensure they can consistently replicate the protocol.

### Sham electro-acupuncture

For sham electro-acupuncture, the K Streitberger placebo needle (Huatuo, Suzhou Medical Supply Factory Co. Ltd., Suzhou, China) will be applied at CV 23 and GV 16. The K Streitberger placebo was invented by Streitberger in 1998, which is a validated and reliable single-blind acupuncture needle used to investigate the effects of acupuncture [24]. This single-blind placebo needle is widely used in acupuncture research. The HANS acupuncture point nerve stimulator will be connected to the placebo. The RMT and MEP of the mylohyoid will be measured before and after electro-acupuncture or sham electro-acupuncture. Previous studies have reported using TMS to study the cortical topography of the human swallowing musculature in healthy subjects by recording RMT and MEP [14].

### Outcome measures

#### Primary outcome measure

TMS has been widely used for assessing cortical excitability. At the same time, the RMT, as a TMS measure, can be used as an indicator of cortical excitability, which is reliable in single pulse TMS measures [25, 26]. Thus, the primary outcome measure will be the percent change in the RMT of the mylohyoid at baseline and after the electro-acupuncture or sham electro-acupuncture treatment. The RMT of the mylohyoid will be delivered by a TMS pulse every 30 s bilaterally to the left and right swallowing motor cortices, respectively, for 10 pulses in the fully relaxed state. The MEP of the mylohyoid should be greater than or equal to 30 μV for at least half, or 5 of the 10 TMS pulses. The RMT is defined as the minimum intensity that is needed to elicit the MEP ≥ 30 μV in at least 50% of pulses.

#### Secondary outcome measures

The secondary outcome measures will be the amplitude (μV) and (ms) of the MEP of the mylohyoid at baseline and after electro-acupuncture or sham electro-acupuncture. The MEP will be induced by a single TMS pulse as a proxy for the TMS evoked potential in this study. The amplitude of the MEP normally changes with the varying intensity of the TMS. The amplitude of the MEP varies greatly as opposed to latency of the MEP. In practical terms, the RMT, which is the minimum intensity of the TMS from each participant, will be detected and recorded first. Then the relative stimulation intensity of the TMS will be calculated by adding or subtracting a percentage of the basic unit of RMT. Commonly, 80–120% RMT is used clinically and in research. In this study, we will use 110% RMT as a relative stimulation intensity to detect the activity of the contralateral mylohyoid swallowing motor cortex every 30 s, for three TMS pulses. In addition, the mean value, in μV and ms, of the three MEP amplitudes and latencies evoked for the contralateral mylohyoid will be recorded.

### Adverse events

All subjects will be assessed before and after the intervention for minor adverse events including fainting, headaches, stuck needles, bent needles, broken needles, and hematomas. If more serious adverse events occur such as hearing impairment or seizures, the trial will be immediately stopped, and necessary medical treatments will be applied. These serious adverse events will be recorded on an adverse event report form and will be monitored by the China Clinical Trial Registration Ethics Committee who may deem that the trial be discontinued.

### Data monitoring and quality control

Risks associated with this study are minimal. The principal investigator, the co-investigators, as well as the acupuncturists administering the intervention are all experienced acupuncturists who can adroitly handle an acupuncture emergency. This team will also ensure that participants consent to the study according to their own free will. The ResMan Research Manager is a public clinical trial data acquisition and management system that was established by the data monitoring committee of the Chinese Clinical Trial Registry. Data will be obtained from the case record form (CRF) and typed into the computer by two data managers. The paper and the electronic data will be checked by the two data managers. The paper data will be stored in a locked file cabinet in the principal investigator’s office at the South China Research Center for Acupuncture and Moxibustion, and the electronic data will be stored in the ResMan Research Manager. The data monitoring committee will be responsible for data monitoring to evaluate safety issues every 6 months. The committee will specifically monitor attrition rates, adverse events, and reasons for attrition and adverse events. All adverse events will be monitored quarterly, but special attention will be given to events requiring medical or surgical intervention that require hospitalization and/or prevention of death and long-term disability. Upon completion of the trial, the electronic data will be examined by the two data managers in the ResMan Research Manager and all subject data will be encoded. Furthermore, the China Clinical Trial Registration Ethics Committee will audit the study randomly as an independent body.

To maintain study quality, the researchers, including acupuncturist and research assistants, will receive specialized training with a test-retest of all trial methods, study techniques, and monitoring methods. The outcome assessment and data analysis study personnel will be blind to the study procedures.

Information on demographics, MMSE, BMI, Kubota Water Swallow Test, outcome data, and reason for attrition will be recorded in the CRF. Subjects may be excluded if they do not meet the inclusion criteria. Participation will be terminated upon subject request or withdrawal of consent. Investigators may also terminate subjects from the study to protect their safety.

### Sample size calculation

The sample size was calculated based on a pretest from a reference in this field, where the sample size was 19.36 and calculated according to the statistical formula: $n=\frac{2\left[{\left(\upalpha +\upbeta \right)}^2{\sigma}^2\right]}{{\left({\upmu}_1-{\upmu}_2\right)}^2}$ where *n* was the sample size of the treatment group/control group, α is the significance level, β is the power of the test, σ is the population variance (SD) and μ_1_ − μ_2_ is the smallest effect of interest. In this study, α = 0.05, β = 0.80 (80%), σ was estimated at 13.07, and μ_1_ − μ_2_ was the RMT of the swallowing-related muscle groups (the most important index to evaluate lateralization of the human swallowing motor cortex excitability). This was the minimum clinical significance value for the RMT of the swallowing muscles that was calculated from previous trials and expert assessments, based on the primary clinical data, which was estimated at 3.57 (Wang, Lin. Effect of Electro-acupuncture at Acupoints on the Swallowing Motor Cortex in Humans, unpublished). Therefore, a sample size of 19.36 is needed in each group (electro-acupuncture and sham acupuncture). We will include 20 subjects per group condition.

Forty healthy subjects will be randomized 1:1 to the electro-acupuncture or the sham electro-acupuncture groups. Both groups will receive TMS (Magstim 200, Magstim Co. Ltd., Whitland, Wales, UK). The RMT and the MEP of the mylohyoid will be recorded using EMG (Nicolet Viking Quest, Wisconsin, USA) before and after one 15-min electro-acupuncture or sham electro-acupuncture treatment. The whole study procedure from assessing eligibility to completion of the treatment will last between 1 and 2 h.

### Statistical analysis

The RMT and MEP statistical analysis will be performed using the Statistical Package for Social Science (SPSS) version 17.0 (SPSS Inc., Chicago, IL, USA). Levene’s test for homogeneity of variance will be applied. If measurement data are normally distributed then the independent *t* test will be used to analyze between-group differences for each subject before and after electro-acupuncture or sham electro-acupuncture, and between the electro-acupuncture group and the sham electro-acupuncture group. Otherwise, the Wilcoxon rank sum test for a nonparametric distribution will be used. A *p* value less than or equal to 0.05 will be considered statistically significant.

### Ethical considerations

This study conforms to the principles of the Declaration of Helsinki. The trial protocol has been approved by the China Ethics Committee of Registering Clinical Trials (reference number ChiECRCT-20170038) on 30 June 2017. Signed consent will be obtained from each subject after they are informed of the procedures, possible risks of the study, the allowances, and their right to discontinue participation at any time. If adverse events occur, the subjects will be treated emergently and the China Ethics Committee of Registering Clinical Trials will be notified.

### Monitoring and publications

The entire progression of the trial and the confidentiality of the data will be monitored by the China Clinical Trial Registration that operates independently from trial researchers and has no competing interests. Any modifications and corrections to operation procedures will be monitored and submitted to the directors of the China Clinical Trial Registration. The findings of the study will be disseminated in academic journals and conference presentations.

## Discussion

### Acupuncture points selection

Ten acupuncture points are used clinically to treat OD. Previous studies suggest that deep needling at Lianquan (CV 23) combined with other swallowing-related acupuncture points such as Jinjin (EX-HN 12) and Yuye (EX-HN 13) effectively improve swallowing function after stroke [27]. According to Traditional Chinese Medicine (TCM) theory, the acupuncture point Fengfu (GV 16) lies on the Governor Vessel meridian, which travels directly to the brain. GV 16 is used to treat brain disorders, including OD. Furthermore, electro-acupuncture stimulation to Fengfu (GV 16) was found to be neuroprotective for patients with OD after stroke by stimulating the downregulation of S100β-mediated neurotoxins [28]. Finally, Fengfu (GV 16) and Lianquan (CV 23) are recommended in the Traditional Chinese Medicine Evidence Based Clinical Practice Guidelines of Acupuncture for the treatment of dysphagia [29]. Therefore, Fengfu (GV 16) and Lianquan (CV 23) will be used for this trial.

### Parameters for electro-acupuncture

The advantage of using electro-acupuncture over manual acupuncture is that the electric current provides continuous stimulation to the needles, which results in a stronger and more effective treatment. Specifically, electro-acupuncture is more effective than manual acupuncture for the treatment of OD [30].

The region of undamaged swallowing motor cortex in OD patients was larger after electrical stimulation [31]. Electro-acupuncture stimulation at particular frequencies to acupuncture points promotes neuroprotective effects against cerebral ischemic reperfusion injury by activating ERK1/2 [32–34]. Different electro-acupuncture stimulation frequencies result in different therapeutic effects [35]. Our preliminary findings demonstrated that the best frequency for electro-acupuncture is 2 Hz with a duration time of 15 min for this current electro-acupuncture intervention [19].

This is the first study to examine swallowing hemispheric lateralization using an innovative combination of TMS, a neuroimaging navigation system, EMG, and electro-acupuncture. Results of this study will determine whether the effects of electro-acupuncture on the human hemispheric lateralization of the swallowing motor cortex excitability exist. We will also begin to describe the influence of electro-acupuncture on the hemispheric lateralization of the swallowing motor cortex excitability. Our eventual aim is to determine the pathological mechanisms involved in OD as a sequela of stroke.

## Trial status

Participants will be recruited for this study from July 2017 to February 2018. The study will be completed by December 2018.

## Additional file

Additional file 1: SPIRIT 2013 checklist: recommended items to address in a clinical trial protocol and related documents. (DOCX 44 kb)

## Abbreviations

BMI: Body mass index
CV: Conception Vessel
EMG: Electromyography
GB: Shaoyang, Gallbladder Channel of Foot
GV: Governor Vessel
LI: Yangming, Large Intestine Channel of Hand
MEP: Motor evoked potential
MMSE: Mini-Mental State Examination
MRI: Magnetic resonance imaging
OD: Oropharyngeal dysphagia
RMT: Resting motor threshold
SPSS: Statistical Package for Social Science
TCM: Traditional Chinese Medicine
TMS: Transcranial magnetic stimulation
